# Adherence timescale impacts completion rates of high-frequency mobile cognitive assessments among older adults

**DOI:** 10.37349/emed.2025.1001356

**Published:** 2025-09-11

**Authors:** Kieffer Christianson, Meha Prabhu, Zachary T Popp, Md Salman Rahman, James Drane, Marissa Lee, Corinna Lathan, Honghuang Lin, Rhoda Au, Preeti Sunderaraman, Phillip H Hwang

**Affiliations:** 1 Department of Psychology, University of Nevada, Reno, NV 89557, USA; 2 Department of Anatomy and Neurobiology, Boston University Chobanian & Avedisian School of Medicine, Boston, MA 02118, USA; 3 AnthroTronix, Silver Spring, MD 20910, USA; 4 Department of Medicine, UMass Chan Medical School, Worcester, MA 01655, USA; 5 The Framingham Heart Study, Boston University Chobanian & Avedisian School of Medicine, Boston, MA 02118, USA; 6 Department of Epidemiology, Boston University School of Public Health, Boston, MA 02118, USA; 7 Department of Neurology, Boston University Chobanian & Avedisian School of Medicine, Boston, MA 02118, USA; 8 Department of Medicine, Boston University Chobanian & Avedisian School of Medicine, Boston, MA 02118, USA; 9 Slone Epidemiology Center, Boston University Chobanian & Avedisian School of Medicine, Boston, MA 02118, USA; 10 Department of Psychiatry and Human Behavior, Brown University, Providence, RI 02912, USA

**Keywords:** remote, digital, cognitive, assessment, high-frequency, adherence, timescale, longitudinal

## Abstract

**Aim:**

Mobile technology enables frequent, remote cognitive assessments, introducing new methodological opportunities and challenges. The study evaluated the feasibility of a high-frequency cognitive assessment schedule among older adults, in terms of total assessments and adherence to a prescribed schedule.

**Methods:**

Thirty-three older adults were recruited from the Boston University Alzheimer’s Disease Research Center (mean age = 73.5 years; 27.3% cognitively impaired; 57.6% female; 81.8% White, 18.2% Black). Participants downloaded the DANA Brain Vital mobile application on their own mobile devices during a remote study visit, and were provided a schedule with seventeen assessments to complete over one year at varying frequencies. The first segment contained three subsegments to be completed within one week, the second segment consisted of weekly subsegments spanning three weeks, and the third and fourth segments consisted of monthly subsegments spanning five and six months, respectively. Three adherence types were defined to reflect incrementally broader adherence timescales: subsegment adherence (strict adherence to each prescribed assessment period), segment adherence (completing the required number of assessments within each broader segment), and cumulative adherence (completing the total number of assessments irrespective of timing).

**Results:**

Completion rates differed depending on the adherence timescale and corresponding adherence type. Using the strictest adherence definition (subsegment adherence), completion rates declined (from 93.9% to 72.7%, *p* = 0.05) during the fourth segment. However, when a broader adherence timescale was applied, completion rates did not decline. Overall completion rates increased as adherence timescale parameters were broadened from subsegment adherence (60.6%) to segment adherence (78.8%), to cumulative adherence (90.9%).

**Conclusions:**

Older adults, including those with cognitive impairment, are able to complete remote cognitive assessments at a high-frequency, but may not necessarily adhere to prescribed schedules. Future high-frequency studies should consider adherence as a potential behavioral variable to complement cognitive test data, while recognizing the potential influence of adherence timescale on interpreting completion rates.

## Introduction

Cognitive disorders are now recognized to result from underlying neurodegenerative processes with an insidious onset, beginning with a prodromal phase during which cognitive functioning may remain intact or only subtly impaired [1–3]. Although various biomarker tests may be utilized to inform diagnostic considerations, the traditional approach for characterizing cognitive symptoms consists of a single session of intensive neuropsychological (NP) testing to determine if generalized impairment and/or disorder-specific patterns of dispersion across cognitive domains are observable [4, 5]. This approach provides reliable estimates of disease at later stages, but does not capture the subtle symptoms that arise sporadically during the early stages of cognitive impairment (CI), in part because standardized NP assessments typically cannot be completed more than once per year and are not structured to detect the subtle cognitive decline inherent to earlier stages of the disease [6]. The subjective experience or observation of these early-stage symptoms can be the very basis for pursuing a formal cognitive evaluation, which in turn is not structured to detect them [7]. During these milder stages of CI, it is often family members or other informants who recognize occasional displays of behavior as irregular relative to an individual’s normative functioning [8–10]. Daily caretaker reports often loosely operationalize behavioral deviations as resulting in an increased frequency of “bad days” [11], although informant reports often do not correspond with an individual’s self-report [12]. A bad day interpreted in the context of an otherwise good week can be considered a fluctuation, whereas a steady increase in bad days over the course of a year represents a gradual decline. A single session of NP testing conducted on a bad day may result in a diagnosis of mild CI (MCI), thus resulting in diagnostic bias. Relatedly, a single session of NP testing conducted on a good day could result in a missed or delayed diagnosis. Waiting at least another year before potentially identifying signs of CI will become increasingly consequential as therapeutics to improve symptoms or delay disease progression are likely to become introduced in the future. Depending on the study design, this may also explain why the reversion from MCI to normal is fairly common [13].

To distinguish between fluctuations and true decline, frequent assessment of cognition and behavior is required to avoid relying on data generated from a single session. Remote digital data collection now addresses the juxtaposition between subjective longitudinal observation and objective single session assessment by enabling objective cognitive data to be collected longitudinally and remotely. Mobile device usage among older adults has increased over the last several years, as 84% of USA adults aged 50+ own a smartphone, and 76% report relying on technology to stay connected with friends and family, according to a 2021 survey [14]. Completing assessments at home, rather than in a formal clinical, setting may also reduce the risk of the “white-coat effect”, which explains reduced memory performance in a clinical environment [15]. Additionally, allowing individuals to complete tasks on their own device as opposed to a study-issued device may contribute to improving the ecological validity of remote assessments [16].

Adherence to the prescribed study schedule consisting of repeated unsupervised assessments represents a novel element of remote study designs, particularly among older adults with CI who may have difficulties remembering to complete assessments according to schedule. Prior studies support the feasibility of cognitively unimpaired adults completing repeated unsupervised assessments via mobile-platform [6, 17–26]. However, evidence as to whether older individuals diagnosed with CI are capable or interested in completing frequent unsupervised mobile assessments remotely is limited [27, 28]. Furthermore, no published studies that we are aware of have considered how the timescale used to define adherence might impact feasibility outcomes, which represents a novel aspect of evaluating self-administered assessments.

The limited research examining adherence and assessment frequency as it relates to cognitive performance may persist for two reasons. The first is that the NP approach traditionally considered cognitive performance to be a relatively stable entity. However, the field now acknowledges that considerable within-person variability may occur when NP tests are repeated over a short period of time [29]. If cognitive performance is conceptualized as a dynamic process, rather than a stable trait, the timescale of assessment becomes important [30]. Identifying the appropriate assessment frequency to correspond with the temporal dynamics of behavior has been discussed among other populations [31], but to our knowledge, it has not been studied in the context of CI. The second possible reason that few studies have examined how assessment frequency impacts cognitive performance could be due to concerns of the ability of older adults with CI to complete unsupervised mobile assessments. Although the feasibility of older adults with CI to complete cognitive assessments at a high-frequency for a period of one-to-two weeks has been demonstrated [27, 28], no study has assessed whether completion rates remain high when assessments are prescribed continuously over a longer period of time. Therefore, this study aimed to determine the feasibility of self-administered mobile cognitive assessments prescribed at varying frequencies among older adults with and without CI using the DANA Brain Vital application over a period of one year.

## Materials and methods

### Study sample

The study sample consisted of participants who were already enrolled in a parent study at the Boston University Alzheimer’s Disease Research Center (ADRC). During their regular annual ADRC visits, participants were asked if they were interested in participating in an additional study involving digital technology. Those who expressed interest were then contacted by our study staff by phone or email. The brief screening agreement was completed electronically while on the phone with a study staff member. Inclusion criteria included not meeting criteria for dementia, owning a mobile device (e.g., smartphone, tablet) with access to the internet, speaking English as a primary language, and being over the age of 40 years at the time of enrollment. CI status was assigned to individuals with MCI according to the National Alzheimer’s Coordinating Center (NACC) Uniform Data Set (UDS) diagnostic criteria version 3 [32, 33], or who scored one-and-a-half standard deviations below the normative mean on NP testing in the absence of functional impairment. All cognitively impaired participants completed the study without the assistance of a study partner. Among potential participants who were contacted but did not enroll, eleven were uninterested, and two did not meet the inclusion criteria.

### DANA brain vital

#### Application description

The DANA Brain Vital tool was utilized as the mobile application in this study. DANA is an FDA-cleared computerized neurocognitive test accessible as a mobile application for iOS and Android devices. Previous work has demonstrated DANA’s internal consistency and reliability [34]. The duration of a single assessment session is approximately five minutes and consists of three subtests intended to measure reaction time, executive functioning, and inhibitory control. The subtests are presented in a fixed order, and performance feedback is not provided to the participant. The first subtest, Simple Reaction Time (SRT), measures the latency between when a large target appears on the screen and when the participant taps the target across 40 trials. The second subtest, Procedural Reaction Time (PRT), involves one of four numbers (2, 3, 4, or 5) being displayed for two seconds and requires the participant to tap the corresponding left button (2 or 3) or right button (4 or 5) across 32 trials. The third subtest, Go/NoGo (GNG), requires the participant to either tap or omit a response based on the color of stimuli that appear in varying locations across the screen across 30 trials [35].

#### Data quality

Data collected from the DANA application is automatically transferred to a secure web portal accessible to the study staff in raw and derived formats. Raw data consists of specific values for each finger tap completed across every trial, whereas summary scores for each subtest within an assessment constitute the derived outcomes that are intended to be readily interpretable. Any subtest with a percentage accuracy of less than 70% is flagged as an indication that the instructions may not have been comprehended or a distraction may have occurred. An additional feature to mitigate extraneous factors influencing test performance is that the application automatically closes if a phone call is received while the application is open. If the application is reopened within three minutes after closing, the assessment may be continued beginning at the subtest that was in progress prior to the interruption. After three minutes of being closed, the data is discarded, and no partial results are recorded.

### Remote study design

Participants were given the option to complete the remote study visit via Zoom videoconferencing or phone. After obtaining informed consent electronically, participants were directed to download the mobile application to their mobile device and log in using a unique study ID. A practice session using the application was completed to ensure that participants were comfortable completing the assessment tasks. Study staff monitored incoming practice data to confirm that assessment task instructions were followed by participants. Study staff were trained using standardized checklists and ensured all participants completed remote consent procedures, successfully downloaded the mobile application, and completed the initial practice session. Participants were provided contact information for study staff and were encouraged to reach out directly with any technical issues or questions that arose after the initial remote study visit.

**Figure 1.**
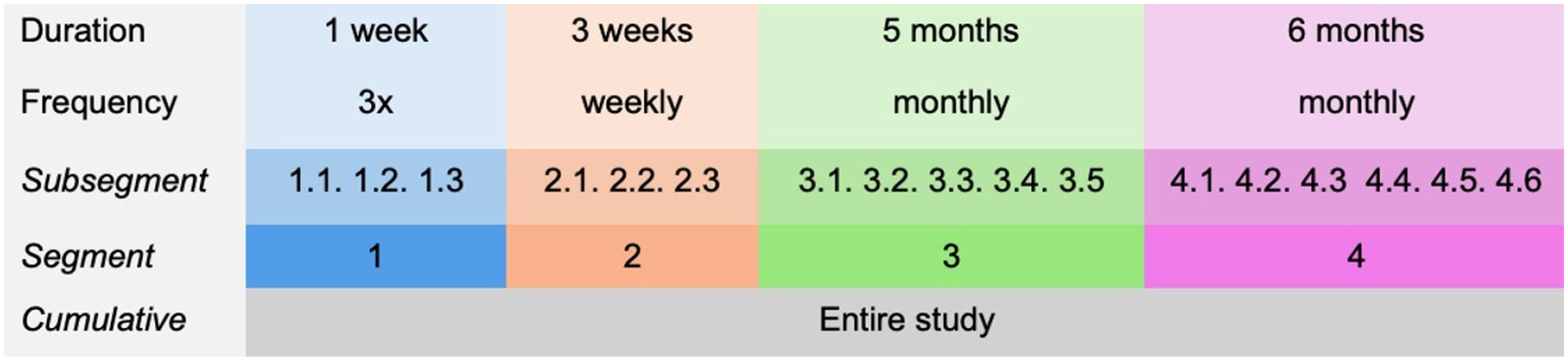
12-Month study schedule. 3x: three assessments in one week, each subsegment is completed one time during the first week.

A self-administered assessment schedule consisting of four segments spanning one year in duration was provided to each participant (Figure 1). Each segment consisted of multiple subsegments, which were defined as specific periods during which individual assessments were expected to be completed. The first segment included three subsegments to be completed within one week; the second segment included weekly subsegments and spanned three weeks; and the third and fourth segments included monthly subsegments spanning five and six months, respectively. Segment durations were varied to inform whether adherence varied as a function of prescribed segment length. A $25 gift card was mailed to participants for each segment completed on time and in its entirety. Participants were only asked to complete the minimum assessment schedule, but were not prevented from completing additional assessments. However, multiple subsegments were not allowed to be completed in one day. Up to three reminders were sent per assessment via text, email, or phone call, depending on participant preference.

### Adherence

**Figure 2.**
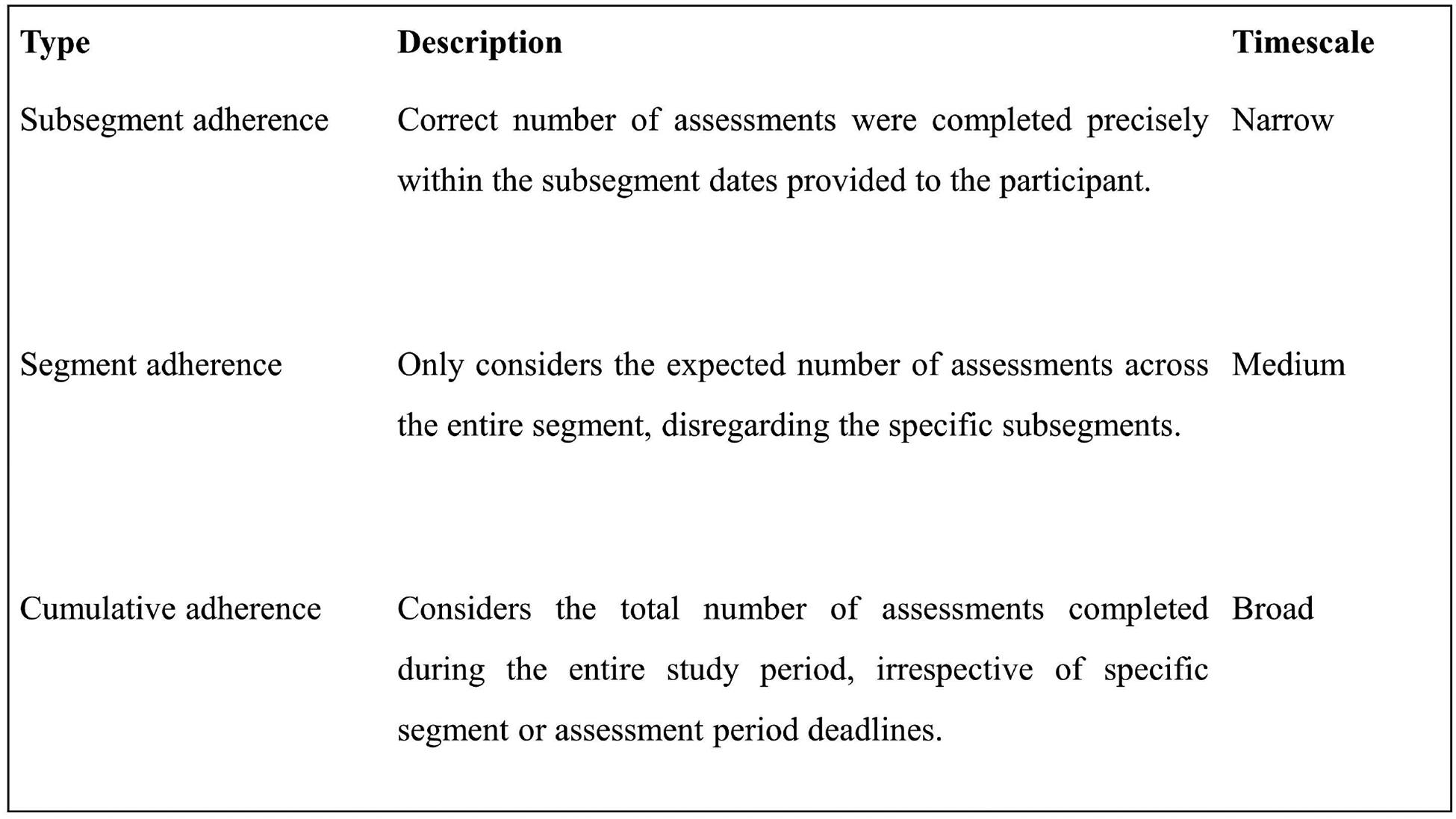
Adherence type definitions.

Feasibility was assessed based on adherence to the study schedule, which was objectively measured by task completion rates across a given study period. However, given the variable and longitudinal structure of the schedule, adherence can be defined differently depending on how narrowly or broadly scheduling parameters are applied, a concept referred to here as the timescale of adherence. To illustrate how adherence outcomes depend upon the chosen timescale, three distinct adherence types corresponding to the schedule structure were defined (subsegment, segment, cumulative; Figure 2). Although these specific adherence types were developed for this study, the broader point emphasized is that adherence outcomes fundamentally depend on the timescale selected for evaluation.

Subsegment adherence refers to whether the correct number of assessments were completed precisely within the subsegment dates provided to the participant. Based on this definition, an assessment completed one day after a prescribed subsegment period would render the entire segment as non-adherent. Segment adherence only considers the expected number of assessments across the entire segment, disregarding the specific subsegments. For example, a participant who did not complete an assessment during subsegment 2.1, but completed two assessments during subsegment 2.2 the following week, and one assessment during subsegment 2.3 would be considered adherent for segment 2 using the segment adherence definition (three assessments within three weeks), but non-adherent using the more stringent subsegment adherence (one assessment each week for three weeks) definition. Both subsegment and segment adherence were evaluated individually across each segment and collectively across the overall study. To meet the overall criteria within either the subsegment or segment adherence type, segment adherence across each individual segment was required. Finally, cumulative adherence considers the total number of assessments completed during the entire study period, irrespective of specific segment or assessment period deadlines. These different definitions of adherence were examined to determine how the temporal resolution of assessment frequency impacts feasibility. Within the scope of this study, the progressively relaxed definitions for adherence provide different insights as to whether participants completed the total number of scheduled assessments, and whether they adhered to the specific schedule separately.

### Analysis

Descriptive statistics for the sample demographics were provided as means and standard deviations for continuous variables or counts and percentages for categorical variables. Adherence rates were computed by dividing the number of participants characterized as adherent within each definition by the total number of participants. Cochran’s Q test was used to evaluate whether there were significant differences in adherence across assessments. Pairwise McNemar’s chi-squared testing with continuity correction was used for post-hoc testing to compare adherence rates between all segments within the subsegment and segment adherence definitions. Statistical significance was based on two-sided *p* < 0.05. All statistical analyses were conducted in R version 4.2.2 (R Foundation for Statistical Computing, Vienna, Austria) [36].

### Ethics

All participants provided informed consent, and the study protocol (H-37474) was approved by the Institutional Review Board of the Boston University Medical Campus. All methods were carried out in accordance with relevant guidelines and regulations. The study was conducted in accordance with the 2024 Declaration of Helsinki.

## Results

The sample consisted of older adults who were predominantly female (*n* = 19, 57.6%), White (*n* = 27, 81.8%), well-educated (mean 16.9 years), and cognitively unimpaired (*n* = 24, 72.7%) (Table 1). Most participants in the study used iPhone mobile devices (*n* = 19, 57.6%) to complete the cognitive assessments in DANA.

Utilizing the strict subsegment adherence definition, the completion rate declined during the study (93.9%, 90.9%, 81.8%, and 72.7% for segments 1–4, respectively). When applying the broader segment adherence definition, the adherence rates remained relatively high across segments (93.9%, 97.0%, 97.0%, and 87.9% for segments 1–4, respectively). Significant differences were observed across segments when comparing across the subsegment adherence completion rates (*p* = 0.04), but not for the segment adherence completion rates (*p* = 0.35). Within the subsegment adherence definition, the segment 4 adherence rate of 72.7% appears lower than the segment 1 completion rate of 93.9% (*p* = 0.05) (Table 2), but did not reach statistical significance which may be attributed to the small sample size. Overall completion rates (Table 2) increased as adherence parameters were broadened from subsegment adherence (60.6%), to segment adherence (78.8%), to cumulative adherence (90.9%).

## Discussion

### Adherence type and completion rates

Our study supports the growing literature that older adults, including those with CI, are capable of completing high-frequency, mobile-based cognitive assessments. We observed that the timescale or stringency by which adherence is defined may also influence completion rates in our sample. This is relevant to the emerging practice of self-administered assessments in that individuals have the latitude to choose when to complete assessments at home or in any quiet environment with internet access, rather than during a scheduled time in a clinical environment. This flexibility highlights an important methodological consideration we introduce as adherence timescale, which emphasizes that objectively measured adherence can vary depending on how scheduling parameters are applied.

Within this present study, the progressive discrepancy between the overall rates for subsegment adherence, segment adherence, and the cumulative adherence indicates that many individuals completed the total number of prescribed assessments, but that they did not complete them according to specific deadlines. In other words, as adherence criteria were relaxed from using specific subsegment dates to considering broader segment dates or the cumulative study duration, participants continued to complete assessments at a consistent rate over a span of one year. This discrepancy between adherence types provides an initial perspective towards how self-administered completion rates may vary based on frequency, such that individuals were less adherent to the specific study schedule as segment durations lengthened and assessment frequency decreased. Also, the overall subsegment adherence rate as compared to the specific subsegment adherence rates indicates that different participants were non-adherent across different segments, suggesting the phenomenon was not isolated to select individuals.

This study supports the feasibility of the high-frequency assessments among older adults, including those with CI, which was previously reported in studies with shorter assessment periods. Among the emerging literature, Nicosia et al., [28] asked participants to complete up to four brief mobile cognitive assessments per day across three seven-day periods spaced six months apart, and found no difference based on CI status and observed an overall adherence rate of 80.42%. Cerino et al., [27] included up to six daily assessments for 16 days, and found only a slight difference in adherence based on CI status wherein the mean completion rate for cognitive unimpaired adults was 85.20%, while the rate for adults with MCI was 78.10%. To the best of our knowledge, ours is the first study to span a complete year with continuous assessments and to compare completion rates according to different assessment frequencies and adherence types.

It is also important to recognize that adherence in remote cognitive studies depends not only on participants’ motivation and cognitive status, but also on the user experience provided by the application and the availability of technical support. In this study, participants had direct access to research staff for assistance with technical issues, which may have contributed positively to adherence rates. An intuitive application design and readily available technical systems are critical when deploying remote cognitive assessments in both research and clinical contexts.

### Enabling process based detection of cognitive impairment

The traditional approach to detecting CI relies on identifying intra-individual variability or dispersion (IIV-D) across cognitive domains [37–39]. This approach compresses item-level responses into subtest scores that are then converted into standard scores to decipher whether significant discrepancy (i.e., dispersion) is observed across cognitive domains. The Boston Process Approach (BPA) was introduced decades ago to address these shortcomings by emphasizing process-based scoring (e.g., characterizing cognitive error types to differentiate disease pathology that would otherwise be indistinguishable using traditional NP summary scores) [40]. Modern adoptions of a process-based approach couple granular digital data with advanced analytics to uncover novel indices of cognitive functioning [40–45]. The enhanced granularity provided by digital assessments enables sensitive detection of disease by capturing fluctuations within individual assessments [27, 46, 47] and across multiple assessment sessions [21, 48, 49].

These modern process-based approaches to cognitive assessment still rely primarily on active participant engagement. Additionally, clinical identification of CI typically involves actively evaluating everyday functioning (e.g., keeping appointments, effectively using everyday technologies) through questionnaires or interviews with individuals and their family members [50]. Meanwhile, innovations in digital health increasingly utilize passive measurement of everyday behaviors to indirectly infer cognitive status [51, 52]. Considering adherence explicitly as a unique variable within high-frequency cognitive assessment protocols could simultaneously yield direct cognitive performance data and indirect insights into participants’ daily functioning. Incorporating adherence could thus conceptually extend the BPA beyond recognizing response patterns alone, to additionally evaluate whether prescribed assessments were completed “on time”. It is our hope that the concept of adherence timescale and the adherence type definitions presented here provide clear parameters for future research, encouraging consideration of adherence timescale as an important variable.

### Limitations

This study provides insight towards future opportunities and challenges associated with remote data collection to assess cognitive functioning, many of which could not be addressed in the study. This study was limited by the small sample size, which affected the interpretations of any statistical comparisons related to feasibility and precluded any comparisons of cognitive performance based on impairment status or other established clinical markers. Furthermore, the generalizability of the results to more diverse populations is restricted as participants were recruited from a clinical research center, consisting mostly of individuals who are White, highly-educated, and inclined towards research participation. It is also acknowledged that the financial compensation received in this study for completing assessments may have influenced adherence. However, the use of financial incentives does not detract from the demonstration that the high-frequency cognitive assessments were feasible. Without financial compensation, it would be difficult to distinguish whether non-adherence reflected inability to complete assessments or lack of motivation. We believe that if high-frequency assessments were integrated into clinical care, individuals may be motivated by potential health benefits rather than external incentives. Assessment reminders provided according to participant preference (i.e., text, email, or phone call) also likely impacted adherence in a fashion that is difficult to directly quantify. However, the relatively homogenous composition of the study sample is likely a reflection of the parent study rather than a function of the study design per se. Conceptually, the remote study design utilized here should enable recruitment of a more representative sample, as has been demonstrated in other literature comparing demographic diversity between in-person and remote research studies [53]. Future efforts should target a larger and more diverse sample, assess both mean scores and variability in cognitive performance relative to assessment frequency, and collect data related to potential within-person influences on cognitive function, such as sleep, diet, stress, and physical activity. Also, while remote assessment may address certain validity risks related to in-person assessment (e.g., white-coat effect), other unique risks specific to remote environments should be recognized. Additionally, future studies may consider counterbalancing segment frequencies by utilizing a crossover design to account for the possibility that some participants may be more adherent to frequent assessment near the beginning of the study.

## Figures and Tables

**Table 1.** Sample characteristics at study enrollment.

Characteristics	Sample (*n* = 33)	
	Mean	SD
Age (years)	73.5	7.0
Education (years)	16.9	2.1
	*n*	%
Sex (female)	19	57.6
**Race**		
White	27	81.8
Black/African American	6	18.2
**Personal mobile device**		
iPhone	19	57.6
Android	11	33.3
iPad	3	9.1
**Cognitive status**		
Cognitively unimpaired	24	72.7
Cognitively impaired	9	27.3

SD: standard deviation.

**Table 2.** Adherence rates across segments and adherence type.

Segment type	Subsegment adherence^a^	Segment adherence^b^	Cumulative adherence
Segment 1	31/33 (93.9%)	31/33 (93.9%)	-
Segment 2	30/33 (90.9%)	32/33 (97.0%)	-
Segment 3	27/33 (81.8%)	32/33 (97.0%)	-
Segment 4	24/33 (72.7%)^c^	29/33 (87.9%)	-
Overall	20/33 (60.6%)	26/33 (78.8%)	30/33 (90.9%)

a: Cochran’s Q Test, *p* = 0.04;

b: Cochran’s Q Test, *p* = 0.35;

c: Pairwise McNemar’s test for segment 1 and segment 4 comparison, adjusted *p* = 0.05.

## Data Availability

Datasets are available on request: the raw data supporting the conclusions of this manuscript will be made available by the authors, without undue reservation, to any qualified researcher.

## Abbreviations

ADRC: Alzheimer’s disease research center
BPA: Boston Process Approach
CI: cognitive impairment
MCI: mild cognitive impairment
NP: neuropsychological
